# Antenatal Corticosteroids in Early and Late Fetal Growth Restriction

**DOI:** 10.3390/jcm14144876

**Published:** 2025-07-09

**Authors:** Valentina Tosto, Carolina Scala, Nicola Fratelli, Anna Fichera, Alessandra Familiari, Ambrogio Pietro Londero, Luca Antonio Ramenghi, Federico Prefumo

**Affiliations:** 1 Obstetrics and Gynecology Unit, IRCCS Istituto Giannina Gaslini, 16147 Genova, Italy; valentinatosto@gaslini.org (V.T.); carolinascala@gaslini.org (C.S.); ambrogiopietro.londero@unige.it (A.P.L.); 2 Division of Obstetrics and Gynecology, ASST Spedali Civili, 25121 Brescia, Italy; nicola.fratelli@asst-spedalicivili.it (N.F.); anna.fichera@unibs.it (A.F.); 3 Department of Clinical and Experimental Sciences, University of Brescia, 25121 Brescia, Italy; 4 Department of Women and Child Health, Women Health Area, Fondazione Policlinico Universitario Agostino Gemelli IRCCS, Università Cattolica del S. Cuore, 00168 Roma, Italy; alessandra.familiari@policlinicogemelli.it; 5 Department of Neuroscience, Rehabilitation, Ophthalmology, Genetics, Mother and Child Health, School of Medical and Pharmaceuticals, University of Genova, 16132 Genova, Italy; lucaramenghi@gaslini.org; 6 Neonatal Intensive Care Unit, IRCCS Istituto Giannina Gaslini, 16147 Genova, Italy

**Keywords:** antenatal corticosteroids, fetal growth restriction, intrauterine growth restriction, early preterm, late preterm, perinatal outcome

## Abstract

Antenatal corticosteroids are widely used to prevent newborn morbidity and mortality in special obstetric circumstances, especially in preterm birth, but there are ongoing concerns about possible neutral or even detrimental short- and long-term effects in pregnancies complicated by fetal growth restriction. Fetuses with growth restriction may be a subset of preterm infants with a particular vulnerability to steroid exposure. The current scientific evidence on exogenous antenatal corticosteroid effects in this population is not conclusive. Gestational age (early versus late) is a critical issue to assess regarding their use as standard care in this special obstetric circumstance.

## 1. Introduction

Antenatal corticosteroid (ACS) treatment has been the keystone therapy for the reduction of neonatal mortality and morbidity in preterm infants since the publication of the first randomized trial, the Auckland Steroid Trial, in 1972 [1]. Since then, many randomized and case–control studies have reported a significant reduction in the major neonatal morbidities that usually complicate preterm birth, in particular respiratory distress syndrome (RDS), intracerebral hemorrhage, and perinatal mortality [2,3]. However, most data reported from these studies fail to assess the effectiveness and the related risks of antenatal use of ACS therapy in high-risk pregnancies complicated by preeclampsia or fetal growth restriction (FGR). The most recent Cochrane review on the topic of antenatal corticosteroids could not make any specific assessment in the subgroup of pregnancies complicated by FGR/SGA [4].

While evidence supports the continued use of a single course of ACSs to promote fetal lung maturation in preterm infants [4], it has yet to be established as to whether corticosteroid treatment is effective or even potentially dangerous in FGR [5,6,7,8]. Moreover, current evidence suggests categorizing FGR into early (up to 32 weeks) and late (from 32 weeks’ gestation) forms, involving different criteria for diagnosis and clinical management [9,10].

It has been reported that ACS treatment leads to temporary fetal behavioral changes: reduced fetal heart rate and a decrease in short-term fetal heart variability (STV) [11]. Moreover, some Doppler studies in pregnancy complicated by FGR showed a temporary decrease in the pulsatility index (PI) of the umbilical artery, modification of the end-diastolic flow in this vessel, and a decrease in the ductus venosus PI. On the contrary, other studies did not confirm these modifications [12,13,14]. Therefore, it is unclear whether these effects might be related to a transient change due to ACSs without fetal sequelae or could represent clinical signs of deterioration of the fetal condition. This is a main issue because fetal growth restriction is associated with an increased risk of preterm birth and, so, FGR fetuses are very likely to be exposed to ACSs. Additionally, only a few studies assessed the effects of ACSs separately in the early or late phenotypes of FGR, where the trade-off between the complications of FGR and the possible beneficial or adverse effects of ACSs is greatly influenced by gestational age at delivery. Although animal model data are also available, they cannot always be linked back to human relevance or differences in pathophysiology, limiting their translational value.

Given these premises, the aim of this review was to investigate the role of ACSs in pregnancies complicated by early and late FGR.

## 2. Methods

We planned a narrative review on the topic of ACS treatment in pregnancies complicated by FGR. Searches were performed in PubMed and Google Scholar, using different combinations of the keywords “corticosteroids”, “steroids”, “betamethasone”, “dexamethasone”, “fetal growth restriction”, and “intrauterine growth restriction”. The reference lists of the retrieved articles were reviewed for possible additional references.

## 3. Findings

### 3.1. Antenatal Corticosteroids in Animal Models of Fetal Growth Restriction

Transient changes in STV within 72 h of the first dose might be due to a direct effect of betamethasone on processes mediated by glucocorticoid receptors. Such receptors are believed to be present in the human fetal brain, including the hypothalamus, hippocampus, and brainstem centers, which are involved in the control of heart activity [11]. According to an alternative hypothesis, suggested by evidence coming from animal studies, baroreceptor-mediated responses to transient elevations in fetal arterial blood pressure induced by antenatal steroids [15,16,17,18] might account for the STV reduction observed. Fetal heart rate variation is known to vary with the development of the human fetal autonomic nervous system, which underlies the observed pattern of changes in the human fetal baseline heart rate, and its short-term and long-term variation, with increasing gestation [19]. The parasympathetic dominance compared to the sympathetic input is known to reduce heart rate variability and to mediate variability during acute hypoxia when parasympathetic control of the fetal heart rate produces a bradycardia, as observed in catheterized animal studies [20]. In sheep models of chronic hypoxia, the gestational age-related increase in fetal heart rate variability is impaired compared to that in control fetuses [19]. An increase in fetal peripheral vascular resistance increases the fetal cardiac after-load and resistance to flow in all fetal circulatory shunts. A transient increase in resistance in the ductus arteriosus increases the right-ventricular end-diastolic pressure, leading to increased right atrial pressure and upstream resistance in the ductus venosus. It could therefore be suggested that baroreceptor-mediated responses to raised peripheral arterial blood pressure could raise vagal tone in an attempt to reduce arterial blood pressure and reduce fetal heart rate variation as a secondary effect. The steroids most commonly used for antenatal administration—betamethasone and dexamethasone—may elicit different molecular and physiological responses; however, such differences are understudied [21].

Animal studies support a positive effect of antenatal corticosteroids on lung maturation, cardiac contractility, and coronary blood flow in models of fetal growth restriction, with no adverse effects on overall fetal weight and brain weight [22]. However, studies on brain development are conflicting [22].

### 3.2. Hemodynamic and Brain Effects of Antenatal Corticosteroid Administration in Growth-Restricted Fetuses

**Figure 1:**
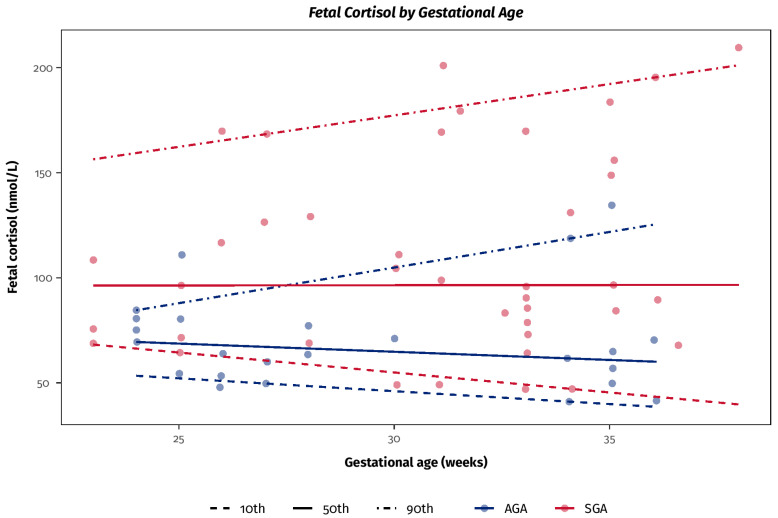
Plasma cortisol concentrations in fetal blood samples obtained from AGA and FGR fetuses. The difference between the medians is statistically significant (*p* < 0.05). Modified from [24].

FGR that occurs before 32–34 weeks of gestation is mainly due to placental disfunction [23] and is therefore associated with chronic fetal hypoxia and raised levels of stress hormones, including endogenous glucocorticoids [24,25] (Figure 1).

FGR may lead to enhanced fetal lung maturation through two main different mechanisms: endogenous cortisol production by adrenal glands due to chronic intrauterine stress, and the downregulation of placental 11-beta-hydroxysteroid dehydrogenase type II (11-bHSD-II) demonstrated in FGR fetuses, increasing exposure to maternal steroids. Assuming these premises, it has been hypothesized that even a single course of ACSs simulates a repeat dose of ACSs, questioning the beneficial role of such treatment in the short and long terms [26].

The cardiovascular adaptations in FGR without exogenous corticosteroids are mainly secondary to a prolonged low-oxygen prenatal environment. These adaptations, which include a “centralization” of blood flow with a preferential redistribution of cardiac output to the brain, are secondary to increased sympathetic activity accompanied by increased secretion of catecholamines [27]. Furthermore, chronic fetal hypoxia causes endothelial vasodilator dysfunction and sympathetic hyperinnervation [28,29]. Modifications in fetal cardiac function in late-FGR fetuses include a more globular left ventricle, an increase in global longitudinal systolic contractility of both ventricles, and increased cardiac output, as well as signs of impaired diastolic function and left-ventricular torsion [30,31].

The administration of ACSs significantly disrupts these cardiovascular modifications in FGR fetuses. It has been demonstrated that maternal administration of betamethasone in pregnancies complicated by FGR determines a significant increase in cardiac output secondary to an increased fetal heart rate and increased stroke volume [32,33]. All these effects are reported only in FGR, while in normal-growth fetuses, the administration of ACSs determines the opposite effects, that is, a decrease in blood flow. Although it is yet to be demonstrated, the massive cardiovascular effect in FGR might reflect decreased systematic vasculature resistance secondary to widespread vasodilatation, particularly in the placenta. Moreover, glucocorticoids are responsible for a selective effect at the cardiac level by increasing nitric oxide synthase (NOS) activity and nitric oxide-mediated dilatation in fetal coronary arteries, and, as mentioned for the increase in blood flow, this effect is seen only in FGR fetuses and not in normal-growth fetuses [34,35].

Concerning the consequences of ACSs on the developing fetal brain in FGR fetuses, limited data are available in the current literature [36]. We have already seen that ACSs in FGR fetuses cause a doubling of cerebral blood flow by increasing cardiac output and cerebral vasodilatation [32].

Healthy and normally grown fetuses respond to prenatal ACSs with a reduction in total cerebral blood flow of about 50%. In contrast, decreased perfusion does not occur in the brain of FGR fetuses, and their total cerebral blood flow increases by over 100%. Experimental data on FGR fetal sheep showed that the increased cerebral vasodilatation is associated with the release of lipid peroxidation products within the brain. Indeed, the increase in cerebral blood flow by over 100% secondary to maternal ACSs in FGR fetuses seems to be associated with evidence of an increase in lipid peroxidation and apoptotic neuronal cell death [33,37]. Therefore, it is possible that exogenous glucocorticoids may be dangerous, leading to further significant neuronal damage, rather than beneficial. A study reported that the ability of the placenta and blood–brain barrier to remove corticosteroids from the fetal compartment or the brain may be compromised in FGR fetuses, with consequent effects on lung, brain, and heart development [38].

### 3.3. Antenatal Corticosteroid Administration in Early-FGR Fetuses

Early FGR may be identified in the group of pregnancies between 24 and 32 or 34 weeks’ gestation, according to available published research [9,10]. The beneficial effects of ACSs in early-FGR newborns on organ system development and clinical outcomes (Table 1) are still unclear.

A prospective, multi-center, population-based study, including 4965 infants born at 24 to 33 weeks’ gestation, investigated the effect of ACSs on neurodevelopmental outcomes according to the head circumference at birth, showing beneficial neurodevelopmental outcomes at 2 years of corrected age in preterm infants with a z score for birth head circumference of >1 SD [41]. Other previous research assessed the effect of ACSs on neurodevelopmental outcomes in preterm and FGR infants. In a single-center retrospective study involving 149 preterm infants with a birth weight below the third percentile and born at 24–32 weeks’ gestation [42], ACSs did not improve neurodevelopmental outcomes. A retrospective Japanese database analysis (Neonatal Research Network Database) evaluated short- and long-term outcomes in infants born with a birth weight of <1500 g and a gestational age between 22 and 33 weeks. A total of 949 infants were evaluated at 3 years: of these, 344 (36%) had received ACSs, and no significant long-term effects were found regarding neurodevelopmental impairment [43]. On the contrary, Schaap et al., in a study on 124 FGR and preterm infants born at 26–31 weeks’ gestation, found a significant protective effect of ACSs on neurodevelopmental outcomes, with greater survival without disability or severe delay at 2 years of corrected age (OR 3.2 [1.1–11.2]) when compared to non-FGR and preterm infants. However, this was a case–control study including a small number of patients from only two centers, and all babies were delivered by Caesarean section [44]. Many of the works in Table 1 are retrospective, single-center studies with small sample sizes and heterogeneous populations. These methodological weaknesses introduce a high risk of bias, limiting the generalizability of the findings and restricting the ability to make causal inferences.

### 3.4. Effect of Antenatal Corticosteroid Administration on Parameters of Fetal Wellbeing

Discordant data have also emerged regarding the effects of antenatal steroids on Doppler parameters. Piazze et al. studied FGR fetuses with absent/reversed umbilical artery end-diastolic flow (EDF) velocity at admission, to evaluate the eventual benefit of antenatal steroid therapy. The restoration of EDF velocimetry in the umbilical artery, improved ductus venosus waveforms, delayed cardiotocographic alterations, and better perinatal outcomes were reported [45].

Another prospective longitudinal multi-center study assessed Doppler flow velocity in fetal, uteroplacental, and maternal arteries before and after ACS therapy among singleton pregnancies complicated by FGR, showing transient improvements in uterine and umbilical artery blood flow among pregnancies affected by growth restriction [46].

On the other hand, Wijnberger et al. observed no effects of antenatal corticosteroids on fetal Doppler waveform patterns of the umbilical artery, middle cerebral artery, and ductus venosus in a cohort of 55 FGR fetuses at 24–34 weeks of gestation [46]. In a smaller cohort, Simchen et al. observed that preterm FGR fetuses with absent/reversed end-diastolic flow exhibited divergent cardiovascular responses to prenatal steroids, hypothesizing that a subset of fetuses may be prone to decompensation after maternal steroid administration [50].

In a rigorously phenotyped cohort of early-FGR fetuses with increased umbilical PI from the TRUFFLE study, Fratelli et al. demonstrated no significant effect of ACSs on ductus venosus PI or on STV at cardiotocography. This suggests that assessments of these two parameters remain valid for the timing of delivery of these fetuses, even in the 48–72 h following ACS administration [51].

Vadivelu et al. assessed changes in fetal Doppler indices and cardiovascular function in pregnancies complicated with FGR between 28 and 36 weeks after the administration of betamethasone: the fetal heart rate, left heart myocardial performance index, and left-sided isovolumic indices showed an improvement after steroid therapy [48].

Marchi et al. prospectively compared appropriate-growth fetuses and FGR singleton pregnancies receiving ACSs for fetal lung maturation between 24^+0^ and 33^+6^ weeks, describing a worsening in cardiac function in FGR group [49].

### 3.5. Timing of Antenatal Corticosteroid Administration in Early-FGR Fetuses

Despite the widespread use of ACSs in perinatal medicine, pharmacokinetic and pharmacodynamic data are scant. Pharmacokinetic models applied to the preterm population suggest that doses of steroids lower than those in current clinical use may be equally effective [52,53]. However, a non-inferiority randomized clinical trial comparing half-dose to full-dose betamethasone demonstrated that the half dose was not non-inferior regarding the primary outcome of the trial, i.e., the need for surfactant use [54], although no difference was found in a secondary analysis for mortality or severe morbidity at discharge [55]. Unfortunately, we could not find any specific pharmacokinetic or pharmacodynamic data in the FGR population.

There is evidence that in the general population of children born preterm after a full course of antenatal steroids, an interval between ACS treatment and delivery of more than 7 days is associated with a lower rate of survival without significant neurologic impairment [56]. However, there are limited data regarding the specific group of early-FGR fetuses. Prins et al. performed a secondary analysis of data from the Dutch STRIDER study, a randomized trial of sildenafil vs. placebo in early severe FGR [57]. Even within the same trial in a single country, they found significant practice variation among institutions, with some centers adopting early ACS administration at the finding of umbilical artery PI at the >95th centile and others using late ACS administration when the umbilical artery showed absent or reverse EDF. Out of 120 pregnancies included, no significant differences were observed in neonatal mortality or composite adverse neonatal outcomes according to the timing of ACS treatment [58].

The early and late ACS administration strategies were compared in a larger multi-center retrospective cohort study of six perinatal centers in the Netherlands [59]. Early and late ACS treatment was administered in 871 and 582 pregnancies, respectively. The interval between ACS administration and delivery was not significantly different between the two groups; however, the late ACS strategy was associated with a non-significant increase in neonatal mortality (adjusted odds ratio 1.47; 95% CI 0.97–2.22). In the whole cohort, delivery took place within the ideal window of 2–7 days from the first ACS injection only in 39% of cases [60]. In the FGR population, the narrow therapeutic windows and variable fetal reserve may hinder the possibility of achieving an optimal interval between the initiation of ACS treatment and the actual delivery. 

Although the soluble fms-like tyrosine kinase-1/placental growth factor ratio, alone [61] or in combination with fetal Dopplers [62], may be useful in predicting the need for preterm birth in FGR, we could not find data regarding its use in the timing of ACS administration.

### 3.6. Antenatal Corticosteroid Administration in Late-FGR Fetuses

Late FGR may be identified in the group of pregnancies between 32 and 36 weeks’ gestation, according to available published research [9,10]. The utility of ACS intervention in women with fetal growth restriction in this gestational period remains unproven [63] (Table 2).

In a retrospective cohort study, Bitar et al. reported that the administration of ACSs in late-preterm-birth FGR pregnancies in the period was not associated with a decrease in composite respiratory outcome [64]. These outcomes may be consistent with several theories suggesting that FGR fetuses are often exposed to a high amount of maternal cortisol and intrauterine fetal stress, leading the fetal adrenal gland to secrete excess cortisol. This may lead to an acceleration of fetal lung maturity at delivery, reducing or nullifying the additional benefit of exogenous steroids.

A most recent multi-center prospective cohort analysis by Familiari et al. did not show a beneficial effect of steroids on short-term outcomes of fetuses with late FGR after 32 weeks’ gestation [26].

Magann et al. reviewed ACS effects on pregnancies between 23–26 weeks and ≥34 weeks’ gestation. Corticosteroid use for early preterms reduces neonatal mortality but not morbidity. In late preterms, ACSs reduce the incidence of respiratory distress syndrome, but there is a lack of follow-up studies on their effects. No significant long-term adverse neurodevelopmental effects have been reported after one or two courses of corticosteroids, but three or more cycles of ACSs are associated with lower overall and organ weights at birth. There may be also an increase in neurodevelopmental abnormalities [6]. A recent systematic review concluded that there is no conclusive evidence on ACSs, even if they are probably beneficial in late FGR [8].

Overall, the literature indicates that the greatest effect of ACSs in the late-FGR group is in the reduction in incidence of transient tachypnea of the newborn, a mostly self-limiting condition. This benefit must be weighed against other adverse outcomes, such as neonatal hypoglycemia, and uncertainties about the long-term neurodevelopmental follow-up and metabolic risks. As a result, the ongoing TRUFFLE-2 trial on the timing of delivery in late FGR did not include any specific recommendation on steroid administration in its protocol, leaving clinical decisions on this issue to the local practice of each participating center [65].

## 4. Limitations

To date, studies have highlighted that well-grown and growth-restricted fetuses display significantly different hemodynamic and hormonal responses to exogenous antenatal glucocorticoids. Although animal studies suggest a benefit of treatment on respiratory and cardiac function, the findings regarding brain development are contradictory [22].

As for human studies, the very first trial on antenatal corticosteroids by Liggins et al. [1] showed a non-significant increase in fetal mortality in cases of severe maternal hypertension and FGR. As a consequence, pregnancies complicated by FGR were excluded from most subsequent trials. Therefore, clinical practice can only be based on observational studies. The several factors complicating direct comparisons among the studies conducted to date include differences in definitions (FGR, RDS), gestational ages, and sample sizes, narrowing the possibility of drawing solid conclusions [66].

Moreover, among growth-restricted fetuses, stratification into early and late FGR [9] may be a critical point for a more balanced rationale regarding the decision to use antenatal corticosteroid treatment in pregnancies before or after 32–34 weeks of gestation [10]. The reported inconsistencies in normally grown versus FGR infants regarding the effect of steroids on neonatal outcomes may be caused by differences in gestational age or the duration of exposure at ACS administration, or by differential effects of glucocorticoids on the development of organ systems.

## 5. Conclusions

Considering the most recent studies, stratification into early FGR and late FGR appears to be a consistent parameter to concentrate on in future research, to study the beneficial, neutral, or harmful effects of ACSs in these special subgroups in order to provide the best management. Table 1 and Table 2 provide data to summarize the current evidence discussed in the text.

ACSs seem to offer a substantial advantage in terms of neonatal mortality and morbidity in early-FGR cases delivered before 34 weeks. However, the evidence in this regard is limited by the heterogenous quality of primary studies and the paucity of information on neurodevelopmental outcomes. After 34 weeks, ACS treatment seems to provide no clear advantage in terms of respiratory or neurological outcomes, and it may expose the newborn to an increased risk of neonatal hypoglycemia; it should therefore be used with caution in this setting.

Given these uncertainties, the decision for and timing of ACS administration in FGR needs to be personalized based on parameters of fetal wellbeing, the timing and mode of expected delivery, fetal reserve, and maternal conditions. There are still numerous unanswered questions, and further research including selected and appropriately FGR-phenotyped cohorts and long-term follow-up is needed to better clarify the real implications of ACS use on lung, cerebral, and cardiovascular development. Ideally, randomized controlled trials stratified by the FGR phenotype, the timing of ACS administration, and long-term neurodevelopmental endpoints should be designed.

## Figures and Tables

**Table 1:** Early FGR and beneficial, neutral, or negative ACS effects observed.

Study	Study Design	Gestational Age (Weeks)	Outcomes
Elimian, 1999 [39]	Retrospective, single-center	Not available (birth weight <1750 gr)	No difference in the incidence of RDS, intraventricular hemorrhage, or necrotizing enterocolitis
Bernstein, 2000 [40]	Retrospective, multi-center	25–30	Beneficial on respiratory distress, intraventricular hemorrhage, neonatal death
Basset, 2018 [41]	Prospective, based-population, multi-center	24–33	Beneficial neurodevelopment effects based on head circumference parameter
Mitsiakos, 2013 [42]	Retrospective, single-center	24–32	No neurodevelopmental effects
Ishikawa, 2015 [43]	Retrospective	22–33	No neurodevelopmental effects
Schaap, 2001 [44]	Case–control	26–31	No neurodevelopmental effects
Piazze, 2012 [45]	Prospective, single-center	28–32	Restored EDF in umbilical artery, improved ductus venosus waveforms, delayed cardiotocographic alterations, better perinatal outcomes
Wijnberger, 2004 [46]	Prospective longitudinal, multi-center	24–34	No effects on fetal Doppler waveform patterns of the umbilical artery, middle cerebral artery, and ductus venosus
Niroomanesh, 2015 [47]	Prospective, longitudinal, multi-center	24–34	Doppler transient improvements in umbilical artery and uterine arteries, no changes in middle cerebral artery flow
Vadivelu, 2021 [48]	Prospective, single-center	28–36	Improved fetal heart indices, especially left-sided isovolumic indices
Marchi, 2020 [49]	Prospective, single-center	24–34	Worsening cardiac function

**Table 2:** Late FGR and beneficial, neutral, or negative ACS effects observed.

Study	Study Design	Gestational Age (Weeks)	Outcomes
Bitar, 2020 [64]	Retrospective, single-center, cohort study	34–36	No benefit from ACSs; increased neonatal hypoglycemia
Familiari, 2023 [26]	Prospective, multi-center observational study	32–36	No benefit from ACSs

## Data Availability

No data available as no new data were generated.

## Abbreviations

The following abbreviations are used in this manuscript:

ACS	Antenatal corticosteroid
AGA	Adequate for gestational age
CI	Confidence interval
EDF	End-diastolic flow
FGR	Fetal growth restriction
PI	Pulsatility index
RDS	Respiratory distress syndrome
